# Antimicrobial and Anti-Inflammatory Activity of Low-Energy Assisted Nanohydrogel of *Azadirachta indica* Oil

**DOI:** 10.3390/gels8070434

**Published:** 2022-07-11

**Authors:** Sukhdeep Kaur, Priyanka Sharma, Aarti Bains, Prince Chawla, Kandi Sridhar, Minaxi Sharma, Baskaran Stephen Inbaraj

**Affiliations:** 1 Department of Biotechnology, CT Institute of Pharmaceutical Sciences, South Campus, Jalandhar 144020, Punjab, India; ksukhdeep380@gmail.com (S.K.); priyanka78660@gmail.com (P.S.); 2 Department of Food Technology and Nutrition, Lovely Professional University, Phagwara 144411, Punjab, India; 3 UMR1253, Science et Technologie du Lait et de l’œuf, INRAE, L’Institut Agro Rennes-Angers, 65 Rue de Saint Brieuc, F-35042 Rennes, France; sridhar4647@gmail.com or; 4 Department of Applied Biology, University of Science and Technology, Ri-Bhoi 793101, Meghalaya, India; 5 Laboratoire de Chimie verte et Produits Biobasés, Département AgroBioscience et Chimie, Haute Ecole Provinciale du Hainaut-Condorcet, 11, Rue de la Sucrerie, 7800 Ath, Belgium; 6 Department of Food Science, Fu Jen Catholic University, New Taipei City 242 05, Taiwan

**Keywords:** phenolic compounds, HPLC, GC–MS, minimum inhibitory concentration, bactericidal, fungicidal, anti-inflammatory

## Abstract

Plant-based bioactive compounds have been utilized to cure diseases caused by pathogenic microorganisms and as a substitute to reduce the side effects of chemically synthesized drugs. Therefore, in the present study, *Azadirachta indica* oil nanohydrogel was prepared to be utilized as an alternate source of the antimicrobial compound. The total phenolic compound in *Azadirachta indica* oil was quantified by chromatography analysis and revealed gallic acid (0.0076 ppm), caffeic acid (0.077 ppm), and syringic acid (0.0129 ppm). Gas chromatography–mass spectrometry analysis of *Azadirachta indica* oil revealed the presence of bioactive components, namely hexadecenoic acid, heptadecanoic acid, ç-linolenic acid, 9-octadecanoic acid (Z)-methyl ester, methyl-8-methyl-nonanoate, eicosanoic acid, methyl ester, and 8-octadecane3-ethyl-5-(2 ethylbutyl). The nanohydrogel showed droplet size of 104.1 nm and −19.3 mV zeta potential. The nanohydrogel showed potential antimicrobial activity against *S. aureus*, *E. coli*, and *C. albicans* with minimum inhibitory, bactericidal, and fungicidal concentrations ranging from 6.25 to 3.125 (µg/mL). The nanohydrogel showed a significantly (*p* < 0.05) higher (8.40 log CFU/mL) value for Gram-negative bacteria *E. coli* compared to Gram-positive *S. aureus* (8.34 log CFU/mL), and in the case of pathogenic fungal strain *C. albicans*, there was a significant (*p* < 0.05) reduction in log CFU/mL value (7.79–6.94). The nanohydrogel showed 50.23–82.57% inhibition in comparison to standard diclofenac sodium (59.47–92.32%). In conclusion, *Azadirachta indica* oil nanohydrogel possesses great potential for antimicrobial and anti-inflammatory activities and therefore can be used as an effective agent.

## 1. Introduction

*Azadirachta indica* seed kernel oil is biodegradable, hydrophobic, and nontoxic, exhibiting a repulsive odor, comprised predominately of the high amount of azadirachtin compound, and various biological properties [1]. *Azadirachta indica* has been established as traditional medicine and employed extensively for agriculture purposes including pest management, nutrient supply, soil quality improvement, nitrification inhibition, soil amendment, and litter compost preparation [2]. The oil also contains several other essential phytocompounds such as salannin, nimbidin, gedunin, nimbin, isomargolone, nimbolide, margolonone, tetranor, terpenoid, lactones, olichinolide-B, azadiradione, meliantriol, desacetyl salanin, and deacetyl nimbin, all exhibiting distinct biological properties including antimicrobial, plasticizer, pesticidal, anti-fertility, mosquito repellent, anti-diabetic, anti-viral, anti-tumor, anti-inflammatory, and antioxidant properties [3,4,5,6,7]. Moreover, the therapeutic potential of the oil has also been established in the treatment of disorders such as periodontitis, vitiligo, ulcers, arthritis, diabetes, and inflammation [8,9]. It is also considered an antifungal agent and used against a variety of pathogenic fungi, including *Alternaria alternate*, *Fusarium moniliform*, *Aspergillus niger*, *Aspergillus flavus*, *Fusarium oxysporum*, *Drechslera hawiiness*, *Fusarium semitectum*, *Curvularia lunata*, *Alternaria solani*, *Sclerotium rolfsii*, *Penicillium expansum*, *Alternaria alternate*, *Monilinia fructicola*, *A. fumigatus*, *Trichothecium roseum*, and *Candida albicans* [2]. The antifungal activity exhibited by *Azadirachta indica* is attributed to the presence of a volatile compound named propyl disulfide [10]. The phytocompounds obtained from plants, however, are much prone to oxidation, due to which notable reduction in bioactivity occurred [11]. The stability and bioavailability of these components can be upgraded using nanotechnology. The high kinetic stability, low optical transparency, viscosity, and the ability to improve the biological activity of lipophilic compounds by escalating the surface area per unit of mass make various nanoformulations an attractive novel system [12]. The presence of arrays of lipophilic, and highly volatile, components in essential oils makes them highly vulnerable to conversion as well as degradation by oxidative and polymerization processes, which results in loss of quantity, flavor, and pharmacological properties [13]. To avoid oxidation as well as loss in the biological activity of *Azadirachta indica* oil, a novel product of nanotechnology, termed nanohydrogels, comprised of oil with an artificial surfactant and emulsifier gum arabic came into fruition. In general, nanohydrogels have a three-dimensional nano-scaled porous structure that exhibits innumerable unique properties including high stability, solubility, biodegradability, and biocompatibility with bioactive compounds [14]. They have a large water-holding capacity and swell up without dissolution due to crosslinking in polymers, which results in their increased surface area. These can be synthesized by various methods; however, the low-energy method is used for the formulation of nanohydrogels as it is the most reliable, cost-effective, more energy-efficient, and it utilizes internal chemical energy, as well as requires simple stirring, thereby allowing the production of a small droplet size. In this process, the formulation of nanohydrogel involves two steps. The first step includes emulsion polymerization using vital components of oil, water, and emulsifier, and the second step involves polymerization of emulsion. The polymerization method contains a homogeneous reaction system in which all monomers, crosslinkers, and initiators are homogeneously dissolved in the same reaction medium before the reaction. Progress in the polymerization reaction results in an increase in the length of the polymer chain. After the growth of the polymer chain to a certain length, the generated phase is separated to form polymer colloidal particles and, finally, nanohydrogels [15]. Because bacteria tend to evolve quickly, they develop resistance to existing antibiotics. This demands other therapeutics as a substitute for antibiotics to deal with bacterial infections [16]. Furthermore, anti-inflammatory agents pose innumerable side effects as therapeutics. For instance, the topical application of anti-inflammatory as well as immune modulators for the treatment of psoriasis leads to the inception of side effects such as cutaneous atrophy and rebound of the disease [17,18]. As mentioned earlier, because *Azadirachta indica* oil constitutes various biological compounds exhibiting antibacterial and anti-inflammatory action, NO-based nanohydrogel may be evaluated for its antibacterial as well as anti-inflammatory potential, to establish its usage as an alternative therapy. The present study is therefore based upon the following objectives: (a) formulation of oil-based nanohydrogels, (b) characterization and development of nanohydrogels, and (c) evaluation of antimicrobial and anti-inflammatory activity of formulated nanohydrogels.

## 2. Results and Discussion

### 2.1. Identification of Phytocompounds

**Figure 1:**
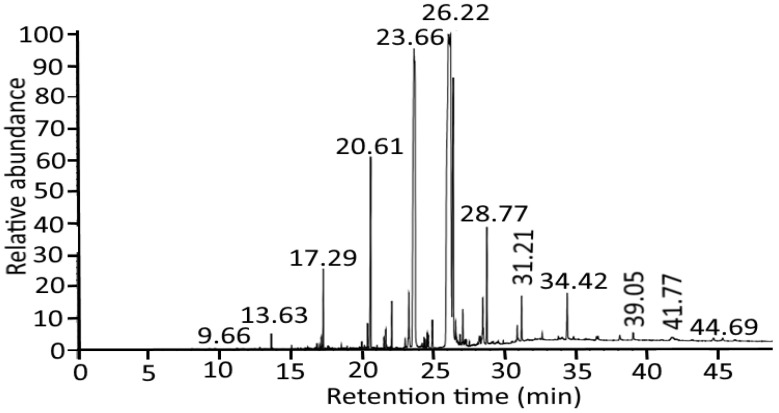
GC–MS chromatogram of *Azadirachta indica* oil showing major bioactive compounds with prominent peaks at different retention times.

The identification of phytocompounds from *Azadirachta indica* oil was carried out by GC–MS analysis, and the results are represented in Figure 1. The chemical profiling of compounds along with retention time and the molecular formula is represented in Table 1. The peaks were recorded at 13.63, 21.54, 23.03, 24.57, 26.08, 28.29, 28.77, 31.21, and 35.74, revealing the presence of Methyl-8-methyl-nonanoate, pentadecanoic acid, hexadecenoic acid, heptadecanoic acid, 9-octadecanoic acid (Z)-methyl ester, 8-Octadecenoic acid, methyl ester, eicosanoic acid, docosanoic acid, 8-Octadecane, and 3-ethyl-5-(2 ethylbutyl). All these compounds have antifungal, anti-influenza, antimicrobial, anti-inflammatory, and antioxidant properties. The present study is in line with Ramalakshi and Shankar [19] and Adigwe et al. [20], who isolated palmitic acid, linoleic acid, oleic acid, octanoic acid, nonanoic acid, hexadecenoic acid, and Methyl-8-methyl-nonanoate from *Azadirachta indica* oil.

### 2.2. Estimation of Phytocompounds by HPLC Analysis

**Figure 2:**
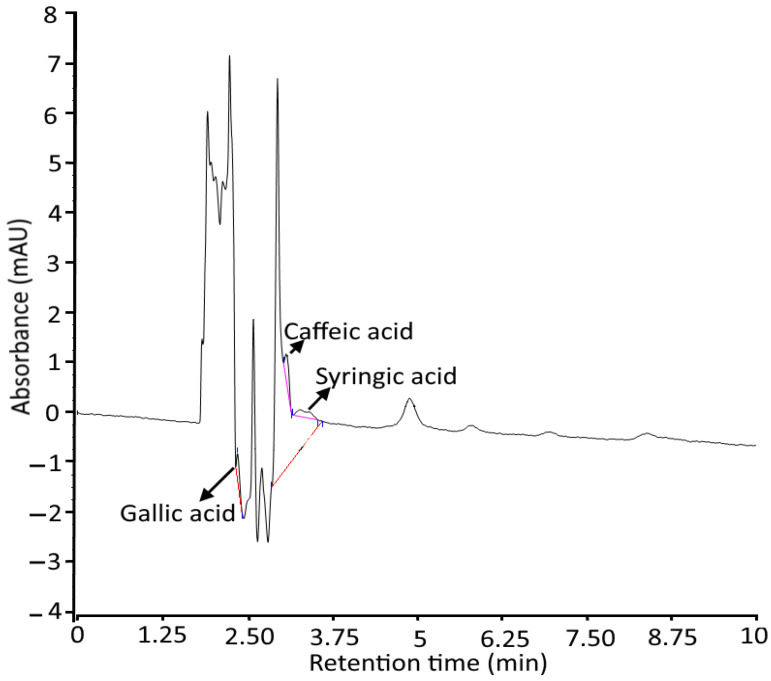
HPLC chromatogram representing quantitative analysis of gallic acid, caeffic acid, and syringic acid in *Azadirachta indica* oil.

The quantification of polyphenols including gallic acid, caffeic acid, and syringic acid was done using the HPLC technique, and the results are represented in Table 2 and Figure 2. According to the results, syringic acid is present in a high amount of 0.0129 ppm, followed by caffeic acid (0.077 ppm) and gallic acid (0.0076 ppm). For each polyphenolic compound, a standard curve was constructed by plotting the concentration of the standard (mg/mL) against the peak area at a specific wavelength. The study represents almost-linear calibration curves through zero points for all samples and internal standards. Our results are in accordance with the findings of Gosse et al. [21] and Cesa et al. [22], and who revealed polyphenolic compounds from *Azadirachta indica* oil.

### 2.3. Characterization of Nanohydrogel

#### 2.3.1. Physiochemical Characteristics of *Azadirachta indica* Oil Nanohydrogels

**Figure 3:**
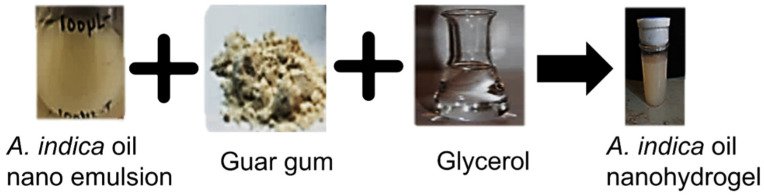
Different stages of nanohydrogel formulation of *Azadirachta indica* oil.

Nanohydrogel was formulated by the emulsion polymerization process including the micellar polymerization of the stabilized *Azadirachta indica* oil emulsion. During the low-energy emulsion preparation process, the appearance of a milky white color confirmed the synthesis of the emulsion containing stabilizer, *Azadirachta indica* oil, and the surface modifier. Furthermore, for micellar polymerization and to formulate the gel, guar gum (gelling agent), pectin (cross-linker), and glycerol (connective linker) were added, and this led to an increase in critical micelle concentration, aggregating in the form of spherical micelles. This resulted in a decrease in the surface tension, and due to this, hydrophobic monomers entered the vicinity of the micelle, and the reaction persisted until all monomer droplets were exhausted and micelle-containing monomers were increased in size. The white-color gel-like structure ensured the formation of *Azadirachta indica* oil nanohydrogel (Figure 3).

#### 2.3.2. Droplet Size Distribution and Zeta Potential of Hydrogels 

**Figure 4:**
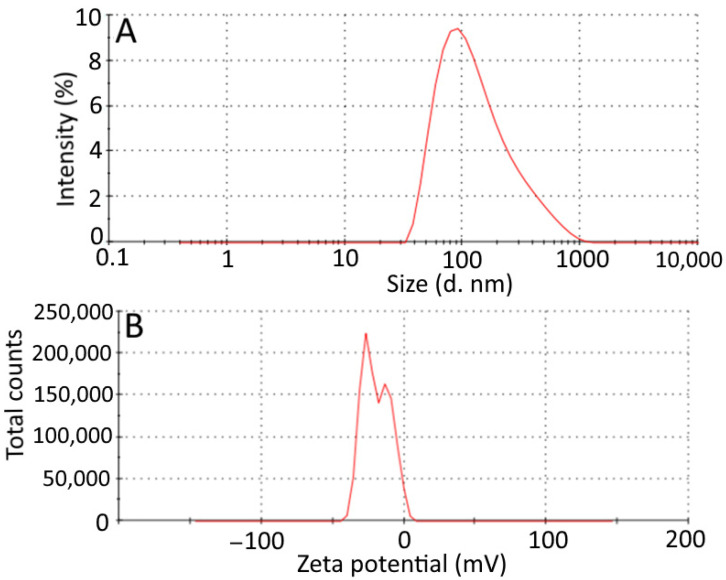
Characteristics of *Azadirachta indica* oil included nanohydrogel. Average droplet size (**A**,**B**) zeta potential.

The average droplet size and zeta potential of *Azadirachta indica* oil hydrogels are depicted in Figure 4A,B. The size distribution curve of *Azadirachta indica* oil nanohydrogel is wider; the average particle size of hydrogel was 104.1 nm. The zeta potential of hydrogel is depicted in Figure 4B; the zeta potential distribution of nanohydrogel was −19.3 mV. The negative charge distribution on the hydrogel sample was due to electrostatic repulsion dominating Van der Waals attractions [23]. The colloidal systems are considered stable, with zeta potential values above 30 mV. The stability is due to sufficient mutual electrostatic repulsion. This principle is effective for surfactants that have low molecular weights and pure electric stabilization and not for high-molecular-weight stabilizers that act by steric stabilization [24,25]. Therefore, in the present study, despite the measured potential value of nanohydrogel, i.e., −19.3 mV, the natural polymer generated the additional steric repulsive force that adsorbed onto the particle surface of the nanohydrogel and enabled it to exhibit good stability, which was in line with findings reported by Wu et al. [26,27].

#### 2.3.3. Differential Scanning Calorimetry

**Figure 5:**
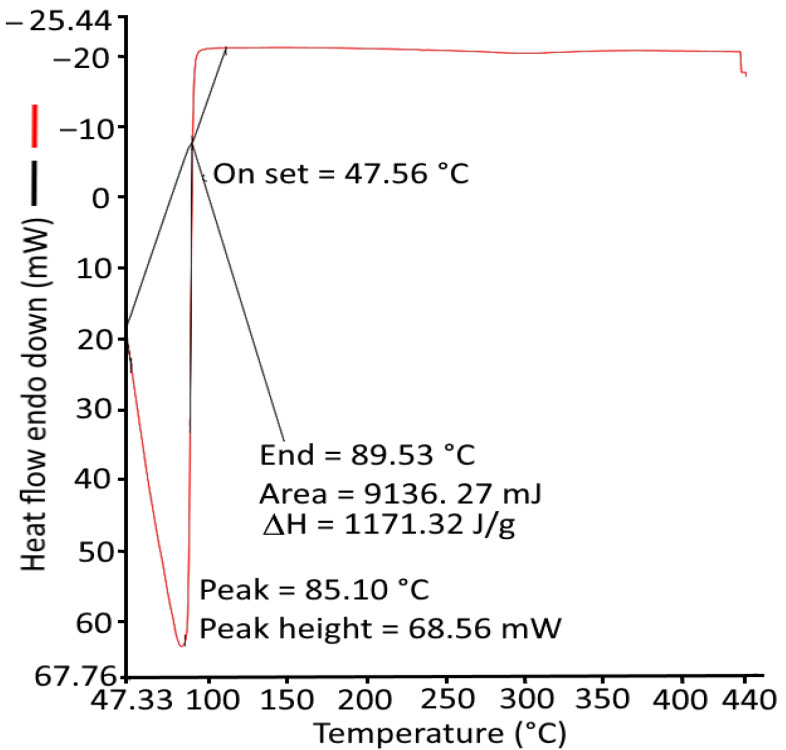
Differential scanning calorimetry spectra of *Azadirachta indica* oil included in the nanohydrogel.

Differential scanning calorimetry was used to study the thermal properties that include identification of changes in phase transition and denaturation (Td) and enthalpy change of nanohydrogels. The results obtained are shown in Figure 5, which summarizes the parameters obtained at a distinct temperature ranging from 50 °C to 440 °C at 10 °C/min for the endothermic reaction at onset temperature (T_onset_) = 47.56 °C, end temperature (T_endset_) = 89.53 °C, denaturation peak temperature (T_denaturation_) = 85.10 °C, and ΔH = 1171.3166 J/g. The onset peak at 47.56 °C can be recognized due to the loss of water that could not be removed from hydrogels upon drying. A similar observation was observed by Thakur et al. [28].

#### 2.3.4. Scanning Electron Microscopy

**Figure 6:**
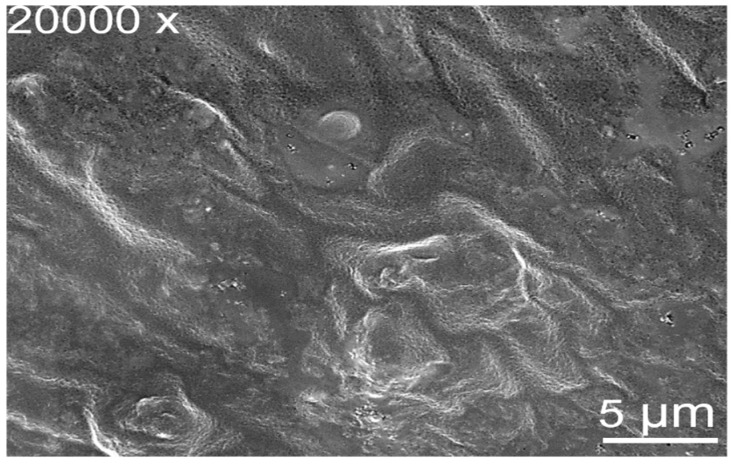
Morphological characteristics of *Azadirachta indica* oil nanohydrogels analyzed by Scanning electron microscope (20,000× magnification).

The surface morphology of *Azadirachta*-*indica*-oil-added nanohydrogel was studied using scanning electron microscopy, and the results are represented in Figure 6. The micrograph was obtained at 20,000× magnification. The micrograph obtained has a clear network and a smooth and dense surface. The dense surface of the nanohydrogel’s membrane limits the passage of microorganisms [29]. The dense surface of the *Azadirachta indica* oil nanohydrogel surface might be explained based on intramolecular hydrogen bonding formation between the surface emulsifier guar gum and and its backbones in nanohydrogel. The present results are in accordance with the studies of Raouf et al. [30] and Khan et al. [31].

### 2.4. FTIR Spectroscopy of Nanohydrogels

**Figure 7:**
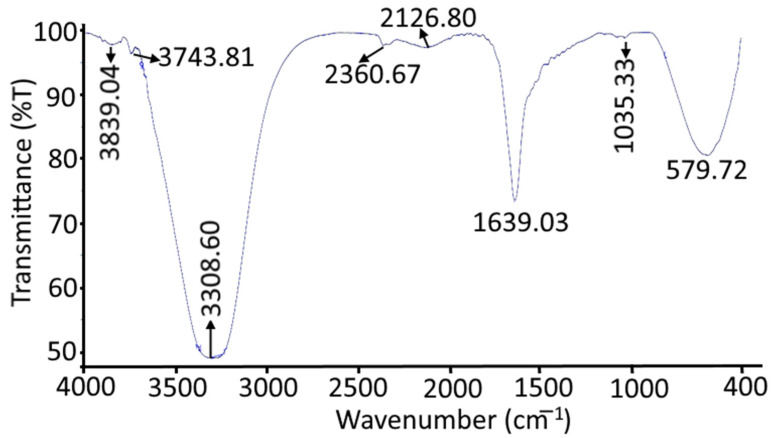
FTIR spectrum of *Azadirachta indica* oil nanohydrogel.

The *Azadirachta indica* oil nanohydrogel sample was subjected to infrared spectroscopy ranging from 4000–400 cm^−1^ based on its functional properties, and the results are represented in Figure 7. The FTIR results elect the occurrence of the hydroxyl group that is responsible for the water-holding capacity in the nanohydrogel membrane [22]. The peak around 3303.60 cm^−1^ represents the hydroxyl group in gum arabic, guar gum, and glycerin. The sharp absorption peak in the ranges of 1639.03 cm^−1^ and 2126.80 cm^−1^ signified the presence of the C=C and C≡C groups, respectively. The absorption peak at 1035.33 cm^−1^ and 579.72 cm^−1^ corresponds to S=O and C–Br stretching. The present results are comparable with the findings of Quereshi et al. [32]. 

### 2.5. In Vitro Antimicrobial Activity

The antimicrobial activity of *Azadirachta indica* oil nanohydrogels against pathogenic bacteria and fungus was expressed as MIC, and the results are represented in Table 3. For pathogenic bacteria, *S. aureus* MIC and MBC values were 6.25 and 3.125; for *E. coli*, the values were 3.125 and 3.125; for *C. albicans*, the values were 62.5- and 6.25-mL *v*/*v*; and for the positive control, MIC and MBC/MFC values for *S. aureus*, *E. coli*, and *C. albicans* were 0.0061, 0.0061 and 0.012, respectively. Therefore, nanohydrogel is endowed with low inhibitory concentration against the growth of *E. coli*, followed by *S. aureus* and *C. albicans*. *S. aureus* is a Gram-positive microorganism that has a thick, porous hydrophobic cell wall. Due to its hydrophobicity, it allows the binding of proteins and lipids, which could be the reason for the high permeability of nanohydrogels. *E. coli* on other hand consists of a cell membrane with membrane-bounded periplasm composed of peptidoglycan that contains teichoic and teichuronic acid. These lipopolysaccharides may lead to a difference in hydrophobic properties and porin mutation that causes resistance of these bacteria against the nanohydrogels [33]. The nanohydrogels have a polycationic nature and also possess a non-stereospecific mechanism that disrupts the cell membrane of pathogenic microorganisms [34]. This characteristic property of nanohydrogels could also be the reason for their antifungal and antibacterial activity in the present study.

#### Time–Kill Kinetics

Time–kill kinetics of neem nanohydrogels were performed, and results are represented in Table 4. Nanohydrogel showed a significantly (*p* < 0.05) higher (8.40 log CFU/mL) value for Gram-negative bacteria *E. coli* compared to Gram-positive *S. aureus* (8.34 log CFU/mL). With the increase in time, nanohydrogel showed a significantly (*p* < 0.05) lower value against *S. aureus* in comparison to *E. coli*. The phenolic compound identified during the characterization of neem results in inhibition of *S. aureus* growth by preventing the biochemical pathway, protein synthesis, and disintegrating of the outer membrane. In the case of Gram-negative *E. coli*, the outer membrane consists of bilayer phospholipid and lipopolysaccharides. These are secure with proteins and β-barrel channels that prevent the penetration of nanohydrogel inside the cell [32]. Besides this, there was a significant (*p* < 0.05) reduction in log CFU/mL value (7.79–6.94) in the case of pathogenic fungal strain *C. albicans*. The reduction in CFU/mL value may be due to the binding of nanohydrogel to the important component of the cell wall that results in pore formation or causes inhibition in the respiration of cells. Another reason for the reduction in CFU/mL value is the interference of nanohydrogel with catalysts and inhibition of synthesis of ergosterol from lanosterol causing cell death [35]. 

### 2.6. Anti-Inflammatory Activity 

**Figure 8:**
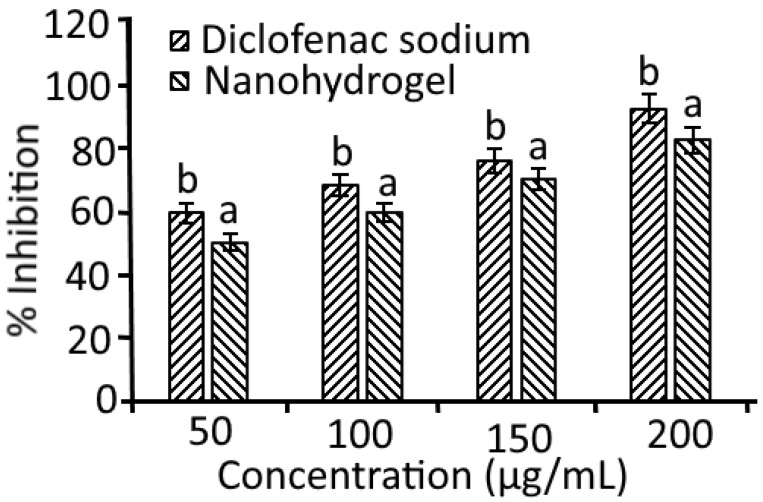
Anti-inflammatory activity of *Azadirachta indica* oil nanohydrogels. Data are presented as means ± SD (n = 3). a,b Means within the column with different lowercase superscripts are significantly different (*p* < 0.05).

An albumin protein denaturation assay was used for the evaluation of the anti-inflammatory properties of *Azadirachta indica* oil nanohydrogels, and the results are depicted in Figure 8. During this assay, a significant (*p* < 0.05) difference was observed in the anti-inflammatory activity of nanohydrogels in comparison to the standard drug diclofenac sodium. The nanohydrogel showed 50.23–82.57% inhibition in comparison to standard (59.47–92.32%). The denaturation of egg albumin protein was induced by a heat treatment that results in the expression of antigens associated with type III hypersensitivity reaction. Type III hypersensitivity reactions are associated with diseases including glomerulonephritis and serum sickness [36]. Similar to naturally occurring proteins, heat-treated denatured proteins can provoke delayed hypersensitivity. In the present study, it has been observed that nanohydrogels showed remarkable anti-inflammatory activity and are proficient to control autoantigen production. Therefore, nanohydrogels can inhibit protein denaturation. During the characterization of *Azadirachta indica* oil by GCMS and HPLC analysis, the secondary metabolites including octadecenoic acid, ç-linolenic acid, methyl ester, syringic acid, and caffeic acid were identified that might be responsible for the anti-inflammatory properties of nanohydrogels in the present study.

## 3. Conclusions

*Azadirachta indica* oil has been utilized to cure severe microbial infections due to the presence of numerous essential bioactive compounds. However, the oil is hydrophobic and hence not easily dispersed in the hydrophilic matrix. Therefore, in the present study, oil-in-water nanoemulsion-based nanohydrogel was formulated. Chromatography analysis revealed the presence of polyphenolic and several other bioactive components. The nanohydrogel showed significantly (*p* < 0.05) enhanced anti-inflammatory activity and also revealed effective antimicrobial activity against both Gram-positive and Gram-negative bacterial pathogenic strains as well as fungal strains. Based on potential results, the developed nanohydrogel can be used for the preparation of topical antifungal and antimicrobial products. It can also be used for the preservation of food materials after scaling up at the industrial level. 

## 4. Materials and Methods

### 4.1. Materials 

*Azadirachta indica* oil was purchased from the local market in Jalandhar, Punjab, India. Tween-40 was procured from HiMedia Laboratories Pvt. Ltd., Mumbai, India. Guar gum, glycerol, and gum arabic were obtained from Sigma Aldrich Co. (St. Louis, MO, USA). Mueller–Hinton agar, Mueller–Hinton broth, Sabouraud dextrose agar, sabouraud dextrose broth, DMSO (dimethyl sulfoxide), streptomycin, phosphate buffer, and sodium chloride were purchased from Hi-Media. Pathogenic bacterial and fungal strains, i.e., *Staphylococcus aureus* (MTCC 3160), *Escherichia coli* (MTCC 443), and *Candida albicans* (MTCC 183) were obtained from the Microbial Type Culture Collection (MTCC), Institute of Microbial Technology, Chandigarh, India. Analytical-grade chemicals and acid-washed glassware were used throughout the experiments.

#### 4.1.1. Identification of Phytocompounds

##### GC–MS Analysis of *Azadirachta indica* Oil

The essential phytocompounds present in *Azadirachta indica* oil were identified and separated using gas chromatography–mass spectrometry (Thermo Fisher Scientific, Waltham, MA, USA). TriPlus RSH autosampler, GC 1300 gas chromatography, TSQ Duo Mass selective quadrupole detector, and TG-5MS column (length 40 m, 0.15 mm internal diameter, and 0.15 µm film thickness) were equipped with the instrument. The samples were diluted in n-hexane with a 1:99 ratio and subjected to analysis. Initially, the GC program was started at 60 °C for 1 min to 180 °C for 3 min, thereby keeping the final temperature at 240 °C for 12 min. For the complete analysis of 34 min, the ramp rate was kept constant at a temperature of 10 °C/min. The helium gas was blown at a constant flow rate of 0.7 mL/mL to run the program. The fragmentation and ionization of separated components were accomplished by electron impact at 70 eV, and the ion source temperature was operated with 250 °C of transfer line temperature at 230 °C. The scanning of the mass filter was set in the range between *m*/*z* 45 and 450. Xcalliber Software was used to process the chromatographic and mass spectra data [33].

##### HPLC Analysis of *Azadirachta indica* oil

The polyphenolic compounds present in *Azadirachta indica* oil were determined using ultra-pressure liquid chromatography (Thermo Fisher Scientific Model: Dionex Ultimate 3000). The system was equipped with a 20RBAX eclipse XDB-C18 column (4.6 × 250 mm, 5 µm, Agilent), and the mobile phase consisted of acetonitrile (70:30) and 1% acetic acid. A stock solution with 1 mL/mL (*V*/*V*) was prepared using methanol, and the sample volume (20 µL) was injected manually in the injector; then, an isocratic run with a flow rate of 1ml/min was employed for 10 min. Diode Array detector (280 nm) with 30 °C column oven temperature was used for the polyphenolic detection and flavonoid detection gradient system consisting of solvent A (water 15, 30, 50, 15%) and solvent B (acetonitrile 85, 70, 50, 85%) with a flow rate of 0.7 mL min^−1^ for 0–20 min. The bioactive compounds present in oil were quantified by using Chromeleon 7.0 software [37].

### 4.2. Preparation of Azadirachta indica Oil Nanohydrogels

The nanohydrogel was prepared by employing the method proposed by Liu et al. [38] with modifications. Briefly, 250 mg of guar gum was dissolved in 250 μL of glycerol and then added to 20 mL prepared *Azadirachta indica* oil emulsion. The resulting reaction mixture was stirred on a magnetic stirrer for 30 min. The formulated *Azadirachta indica* oil nanohydrogel was kept at room temperature for further analysis.

### 4.3. Characterization of Nanohydrogels

#### 4.3.1. Droplet Size Distribution and Zeta Potential of Hydrogels

Dynamic light scattering and laser doppler microelectrophoresis techniques were used to evaluate the droplet size distribution and zeta potential of the hydrogel. Accordingly, 0.1 mL of each sample was dissolved in phosphate buffer (0.05 M) and kept at pH 7 before analysis. The analysis was carried out at 25 °C using a droplet size and zeta potential analyzer (Zetasizer Nano ZS, Malvern Instruments Ltd., Malvern WR14 1XZ, UK) [14].

#### 4.3.2. Differential Scanning Calorimetry

The physiochemical interactions among different components within the *Azadirachta indica* oil nanohydrogel were examined and estimated by differential scanning calorimetry (DSC) using a differential scanning calorimeter (Perkin Elmer DSC 6000, Ueberlingen, Germany). The experiment was performed in an atmosphere with 99.999% nitrogen gas and a rise in temperature from 0.01 °C min^−1^ to 100 °C min^−1^. The thermal protocol was initiated at 10–450 °C at a rate of 10 °C min^−1^ to record the measurements. The plot results were determined by using thermocouple-based temperature sensors.

#### 4.3.3. Field Emission Scanning Electron Microscopy (FESEM)

Field emission scanning electron microscopy (FEI QUANTA 250 Field Emission Scanning Electron Microscope, FEICO, Hillsboro, OR, USA) was used for the evaluation of emulsion and hydrogel samples. Briefly, small amounts of both samples were fixed on a carbon-coated copper grid and kept for drying at 200–30,000 volts for about 10–15 min before imaging [39].

#### 4.3.4. Fourier Transform Infrared (FTIR) Spectroscopy

The functional group present in the *Azadirachta indica* oil nanohydrogel sample was identified by FTIR spectroscopy (Perkin Elmer, Spectrum 2, Ueberlingen, Germany). The data were obtained in the mid-infrared spectral region 4000–600 cm^−1^ in terms of transmission using air as background [14].

### 4.4. Anti-Inflammatory Activity of Nanohydrogels

#### 4.4.1. Antimicrobial Activity

##### In-Vitro Minimum Inhibitory Concentration (MIC), Minimum Fungicidal Concentration (MFC), and Minimum Bactericidal (MBC), of Nanohydrogels

The MIC, MBC, and MFC of *Azadirachta indica* oil nanohydrogels were studied by using the microdilution method given by the Clinical and Laboratory Standard Institute (CLSI) with minor modifications [40]. Herein, a 2-fold dilution of nanohydrogel of different concentrations ranging from 50 to 1.56% mL *v*/*v* was prepared in a microdilution assay plate. Mueller–Hinton broth (MHB) and Sabouraud dextrose broth (SDB) were used as test mediums for the inoculation of pathogenic bacterial and fungal strains, respectively. The microtiter plates inoculated with bacterial strains were incubated at 37 °C for 24 h, and the microtiter plate inoculated with fungal strain was incubated at 27 °C for 48 h, and after incubation, the visual readings were performed. Minimum bactericidal concentration was determined by subculturing the MHA and SDA agar plates. Antibiotic streptomycin was taken as positive control.

##### Time–Killed Kinetics

A kinetic study was executed by using the method followed by Najda et al. [33]. The pathogenic bacterial strains were inoculated in a test tube containing Mueller–Hinton broth, and the fungal strain was inoculated in a test tube containing Sabouraud dextrose broth. The bacterial samples were observed after time intervals of 0, 24, and 48 h, and the fungal strain was observed after intervals of 0, 24, 48, and 72 h. The results were obtained by calculating log CFU/mL for each sample.

### 4.5. Albumin Denaturation Assay 

The anti-inflammatory effect of *Azadirachta indica* oil nanohydrogels was evaluated by using an albumin denaturation assay. Herein, fresh egg albumin (0.2 mL) and phosphate buffer solution (2.8 mL pH 6.4) was mixed with 2 ml of each sample to make a volume of 5 mL. The reaction mixture was incubated in a BOD incubator for 15 min at a temperature of 37 °C. After incubation, the absorbance for each sample was observed at 660 nm using a spectrophotometer. Diclofenac sodium salt and double-distilled water were used as positive and negative controls, respectively [37]. Percentage inhibition of albumin denaturation assay was calculated using the formula:

$$Inhibition\left(\%\right)=100\times (\frac{Ar}{Ap}-1)$$

where *Ar* is the absorbance of the test sample and *Ap* is the absorbance of the control.

### 4.6. Statistical Analysis

For the statistical analysis, the method proposed by Kaushik et al. [41] was employed. The significant difference among the samples was calculated by one-way analysis of variance (ANOVA), and the comparison between means was calculated by critical difference (CD value). Microsoft Excel, 2019 (Microsoft Corp., Redmond, WA, USA) was used for the calculation of means and standard deviation. 

## Figures and Tables

**Table 1:** Potential bioactive components of *Azadirachta indica* oil revealed by GC–MS analysis.

Compound Isolated	Retention Time	Molecular Formula
Methyl-8-methyl-nonanoate	13.63	C_11_H_22_O_2_
pentadecanoic acid	21.54	C_16_H_32_O_2_
hexadecenoic acid	23.03	C_17_H_34_O_2_
heptadecanoic acid	24.57	C_18_H_36_O_2_
9-octadecanoic acid (Z)-methyl ester	26.08	C_19_H3_6_O_2_
ç-Linolenic acid, methyl ester	28.29	C_19_H_32_O
eicosanoic acid, methyl ester	28.77	C_21_H_42_O_2_
docosanoic acid, methyl ester	31.21	C_23_H_46_O_2_
8-Octadecane		
3-ethyl-5-(2 ethylbutyl)	35.74	C_26_H_54_

**Table 2:** Quantification of phytocompounds by HPLC analysis.

Phyto Compound	Retention Time (min)	Quantity (mg/kg)
Gallic acid	2.350	0.0076
Caffeic acid	3.087	0.077
Syringic acid	3.273	0.0129

**Table 3:** MIC, MBC, and MFC of nanohydrogels against pathogenic bacteria and fungi.

Microorganism	MIC mL *v*/*v*	MBC/MFC mL *v*/*v*	POSITIVE CONTROL (Streptomycin) MBC/MFC mL/*v*/*v*
*S. aureus*	6.25	3.125	0.0061
*E. coli*	3.125	3.125	0.0061
*C. albicans*	6.25	6.25	0.012

**Table 4:** Time–kill study of nanohydrogel against *Escherichia coli*, *S. aureus*, and *Candida albicans*.

Time (h)	*E. coli* (Log CFU/mL)	*S. aureus* (Log CFU/mL)	Time (h)	*C. albicans* (Log CFU/mL)
**0**	8.40 ± 0.32 ^a^	8.34 ± 0.28 ^a^	**48**	7.79 ± 0.32 ^d^
**18**	8.27 ± 0.22 ^b^	8.18 ± 0.36 ^a^	**72**	7.32 ± 0.46 ^c^
**24**	8.14 ± 0.19 ^b^	7.94 ± 0.52 ^a^	**96**	7.10 ± 0.21 ^b^
**48**	7.98 ± 0.28 ^b^	7.83 ± 0.50 ^a^	**120**	6.94 ± 0.44 ^a^

Data are presented as means ± SD (n = 3). ^a–d^ Means within the column with different lowercase superscripts are significantly different (*p* < 0.05) from each other.

## Data Availability

No new data were created or analyzed in this study. Data sharing is not applicable to this article.
